# Identification and comparison of novel circular RNAs with associated co-expression and competing endogenous RNA networks in postmenopausal osteoporosis

**DOI:** 10.1186/s13018-021-02604-1

**Published:** 2021-07-16

**Authors:** Weiyi Diao, Yongguang Wang, Jun Zhang, Haiyu Shao, Yazeng Huang, Mengran Jin

**Affiliations:** 1 Department of Orthopedics, The First Affiliated Hospital of Zhejiang University, Qingchun Road No. 79, Hangzhou, 310001 Zhejiang Province China; 2 Department of Orthopedics, The Fifth People’s Hospital of Yuhang District, Baojian Road No. 60, Hangzhou, 310013 Zhejiang Province China; 3 Department of Orthopedics, Zhejiang Provincial People’s Hospital, Shangtang Road No. 158, Hangzhou, 310014 Zhejiang Province China

**Keywords:** Postmenopausal osteoporosis, circRNAs, Competitive endogenous RNA, Whole transcriptome sequencing

## Abstract

**Background:**

Circular RNAs (circRNAs) are emerging as crucial regulators in various human diseases. So far, the expression profile and regulatory mechanism of circRNAs in postmenopausal osteoporosis (PMOP) are less studied and should be deciphered urgently. Herein, we aimed to reveal key circRNAs affecting PMOP and clarify their compounding regulatory actions.

**Methods:**

To reveal key circRNAs affecting PMOP and clarify their compounding regulatory actions, whole transcriptome sequencing and bioinformatics analysis were performed to identify differentially expressed circRNAs (DECs). The expression pattern and regulatory networks of DECs in peripheral blood mononuclear cells (PBMCs) were unearthed.

**Results:**

A total of 373 DECs comprising 123 intronic, 100 antisense, 70 exonic, 55 intergenic, and 25 sense-overlapping circRNAs were identified. Among these, 73 circRNAs were upregulated and 300 were downregulated. These DECs exerted pivotal functions in the pathogenesis of PMOP as demonstrated by Gene Ontology (GO) annotation and the Kyoto Encyclopedia of Genes and Genomes (KEGG) pathway analysis. The circRNA-miRNA-mRNA co-expression network comprising 28 DECs, 145 miRNAs, and 175 differentially expressed mRNAs predicted the possible mechanism of the pathogenesis and progression of PMOP.

**Conclusion:**

The results of the present study provided a further comprehension of circRNA-associated competing endogenous RNA regulatory mechanism in PMOP. The steadily expressed and disease-specific DECs may serve as promising diagnostic and prognostic biomarkers for PMOP.

**Supplementary Information:**

The online version contains supplementary material available at 10.1186/s13018-021-02604-1.

## Introduction

Osteoporosis, a pervasive public health concern worldwide, manifests as the depletion of bone mineral with structure deterioration of bone tissue and results in a predisposition to fragility fractures, especially in women [1]. National registry statistics show that osteoporosis affects approximately 40.1% of all postmenopausal women in China and the resulting fragility fractures are associated with significant morbidity, mortality, and financial implications [2]. To alleviate the public health burden of postmenopausal osteoporosis (PMOP), it is a prerequisite to screen out the population at high risk for fracture, which entails the identification of more rapid, specific, and sensitive bone turnover markers (BTMs) [3].

Circular RNAs (circRNAs), a relatively new class of non-coding RNAs (ncRNAs), form a continuous cycle of covalent closures and are highly expressed in the eukaryotic transcriptome [4]. Accumulating evidence has revealed the regulatory roles of circRNAs, such as functioning as microRNA (miRNA) sponges, serving as scaffolds in the assembly of protein complexes, regulating the expression of parental genes, and modulating alternate splicing, as well as RNA-protein interactions [5–9]. Moreover, circRNAs are expressed more steadily and abundantly than the standard linear transcription of homologous genes due to the closed-loop structure, which prevents degradation by RNA exonuclease [8]. In this way, disease-specific and tissue-specific circRNAs could act as biomarkers for the early diagnosis and prediction of certain diseases theoretically [10]. So far, circRNAs are demonstrated to be expressed in a tissue specific manner and regulate various pathophysiological events, such as organogenesis, tumorigenesis, and organ development [11–14]. However, specific and sensitive circRNA biomarkers for PMOP have not been fully established. Herein, the present study aimed to uncover accurate circRNA biomarkers and provide clues for exploring the underlying mechanism of PMOP. With the aid of whole transcriptome sequencing, differentially expressed circRNAs (DECs) between PMOP patients and normal controls were identified. Functional annotation and protein-protein interaction network constructions were also performed to explore the biological functions of targeted DECs. These findings will help elucidate the mechanism by which circRNA regulates the balance between osteogenesis and osteoclastogenesis. Moreover, the present study is foundational for subsequent studies on circRNAs in PMOP and may offer insight into prevention and new treatment targets for PMOP.

## Materials and methods

### Ethics statement

This study was approved by the Medical Ethics Committee of Local Institution. Written informed consent was obtained from each participant before enrollment.

### Subjects

Postmenopausal patients who received percutaneous kyphoplasty (PKP) surgery or bone mineral density (BMD) examination in the clinic department of our institution between December 2019 and January 2020 were evaluated. Participants were enrolled in this study using the following inclusion and exclusion criteria. The inclusion criteria included (1) age between 55 and 65 years old; (2) at least 1 year after natural menopause; (3) T-scores < −2.5 standard deviation SD (PMOP group) or T-scores > 0 SD (control group) at their lumbar vertebrates [3]; (4) received lumbar PKP surgery (PMOP group) or without PMOP-associated fractures (control group). The exclusion criteria included (1) secondary osteoporosis due to metabolic, blood, thyroid, tumor, drug, and nutritional disorders; (2) premature menopause less than 45 years old; (3) patients who received ovariectomy; (4) had taken calcium, vitamin D, bisphosphonates, and estrogen in the past 3 months. Finally, three paired fresh venous blood samples were collected for whole transcriptome sequencing and 30 paired samples were used for quantitative evaluation using real-time quantitative polymerase chain reaction (qRT-PCR). The demographic and clinical characteristics of the six participants are summarized in Table 1.

**Table 1:** Demographic characteristics of the participants

	PMOF				Control				*P* value
Items	1	2	3	Mean ± SD	1	2	3	Mean ± SD	
Age (years)	55	59	64	59.33± 4.51	55	55	57	55.67± 1.15	0.18
Height (cm)	154	162	160	158.66 ± 4.16	162	166	158	162.0 ± 4.0	0.31
Weight (kg)	52	57	53	54.00 ± 2.65	58	61	51	56.66 ± 5.13	0.22
T-score	−3.2	−3.5	−2.9	−3.20 ± 0.30	1.1	0.9	0.4	0.80 ± 0.36	.003**
PINP (ng/ml)	71.52	79.33	87.49	79.44 ± 7.99	47.62	51.83	41.37	46.94 ± 5.26	.000**
β-CROSSL (ng/ml)	0.81	0.76	0.72	0.76 ± 0.04	0.44	0.58	0.52	0.51 ± 0.07	.001**
Osteocalcin (ng/ml)	28.71	25.42	25.17	26.43 ± 1.98	18.75	17.38	21.36	19.16 ± 2.02	0.02*

*PMOF* postmenopausal osteoporotic fracture, *PINP* type I procollagen N-terminal propeptide, *β-CROSSL* β cross-linked C-telopeptide of type I collagen

^*^Values were compared between the PMOF and control groups, and the result was statistically significant (*P* < 0.05)

^**^Values were compared between the PMOF and control groups, and the result was statistically significant (*P* < 0.01)

### Isolation of peripheral blood mononuclear cells (PBMCs)

Early in the morning, fresh venous whole blood was collected from each participant in a 2.5 ml PAXgene tube (BD, Franklin Lakes, NJ, USA), followed by the isolation of PBMCs within 6 h of collection. PBMCs were isolated at room temperature (18-20 °C) using Ficoll-Paque PLUS reagent (GE Healthcare, Piscataway, NJ, USA) according to the manufacturer’s instruction.

### RNA isolation

Total RNA was extracted from the PBMCs and enriched using Trizol reagent (Invitrogen, Carlsbad, CA, USA) according to the manufacturer’s instruction. RNA quality and quantity were evaluated on a Nanodrop spectrophotometer (ND-1000, Thermo Fisher Scientific, Waltham, MA, USA). RNA integrity and genomic DNA (gDNA) contamination were determined by gel electrophoresis.

### Whole transcriptome sequencing

RNA high throughput sequencing was performed Cloud-Seq Biotech Ltd. Co. (Shanghai, China). Briefly, total RNA was used after removing ribosomal RNAs (rRNAs) with NEBNext rRNA Depletion Kit (New England Biolabs, Inc., Massachusetts, USA) and constructing RNA libraries with NEBNext® Ultra™ II Directional RNA Library Prep Kit (New England Biolabs, Inc., Massachusetts, USA). Libraries were controlled for quality and quantified using the BioAnalyzer 2100 system (Agilent Technologies, Inc., USA). Libraries (10 pM) were denatured as single-stranded DNA molecules, captured on Illumina flow cells, amplified in situ as clusters and finally sequenced for 150 cycles on the Illumina HiSeq sequencer (HiSeq 4000; Illumina, Inc.).

### RNA sequence analysis

Paired-end reads were harvested from Illumina HiSeq 4000 sequencer and quality controlled by Q30 (*P* < 0.001). After 3′ adaptor trimming and low-quality reads removal using the cutadapt software (version 1.9.3), high-quality trimmed reads were aligned to the reference genome/transcriptome (UCSC hg19) guided by the Ensembl Gff gene annotation file with the HISAT2 software (version 2.0.4) and the STAR software (version 2.5.1b). Detection and identification of circRNAs were performed with the DCC software (version 0.4.4). The edgeR software (v3.16.5) was used to normalized the data and obtain the expression profiles of circRNAs in terms of the fragments per kilobase of transcript per million fragments mapped (FPKM) and the cuffdiff software (version 2.2.1) for the expression profiles of mRNAs. Subsequently, the fold-change (FC) and *P* value were calculated based on FPKM. DECs were compared between the PMOP and the control groups following the criteria of |log(FC)| ≥ 2 and *P* value < 0.05. Differentially expressed mRNAs (DEMs) were compared between the PMOP and the control groups following the criteria of |log_2_(FC)| ≥ 2 and *P* value < 0.05.

### qRT-PCR

After isolating and enriching the RNA as aforementioned, 8 circRNAs were selected and validated using qRT-PCR. Total RNA was reverse transcribed into cDNA using SuperScript III reverse transcriptase (Invitrogen; Thermo Fisher Scientific, Inc.). Then, a 2X PCR master mix (CloudSeq Biotech, Inc., Shanghai, China) was utilized to proceed with the qRT-PCR with selected RNAs and internal reference, β-actin (ACTB) of the samples. Primers for each circRNA were designed with the CircPrimer software (version 1.2) and listed in Supplementary Table 1.

The program was initiated by denaturation at 95 °C for 1 min, and thermal cycling conditions were set to 40 cycles at 95 °C for 15 s and 60 °C for 30 s. Data were analyzed using the 2^−∆∆Ct^ method.

### Functional group analysis

Database for Annotation, Visualization and Integrated Discovery (DAVID), a bioinformatics web server (https://david.ncifcrf.gov/), was referred to explore the potential functions of the liner transcripts [15]. Gene Ontology (GO) analysis constituted with three domains, namely, the “biological process,” “cellular component,” and “molecular function,” was performed. The pathway-enrichment analysis was also performed according to the Kyoto Encyclopedia of Genes and Genomes (KEGG) pathways.

### Target miRNA prediction

The circMic software (http://www.bioinf.com.cn/) was referred to predict specific microRNAs (miRNAs) of corresponding DECs. Interactions between miRNAs and circRNAs were evaluated with miRanda (http://www.microRNA.org/). The interaction between dysregulated mRNA and miRNA were predicted with TargetScan release 7.0 (http://www.targetscan.org/) and presented via miRNet 2.0 platform (https://www.mirnet.ca/).

### circRNA-miRNA-mRNA network prediction

The overlapping miRNAs between the predicted circRNAs target miRNAs and the predicted miRNAs targeting the DEMs were adopted for further analysis. miRNA binding sites were predicted with miRcode (http://www.mircode.org/). Finally, the circRNA-miRNA-mRNA regulatory network was constructed using a combination of circRNA-miRNA pairs and miRNA-mRNA pairs and visualized using the Cytoscape 3.6.0 software.

### Statistical analysis

GraphPad Prism version 5.0 (GraphPad Software, San Diego, CA, USA) was used to perform a two-tailed independent *t* test or analysis of variance (ANOVA) for data processing. Results with *P* values < 0.05 were considered statistically significant.

## Result

### Identification of DECs between PMOP and control individuals

**Fig. 1:**
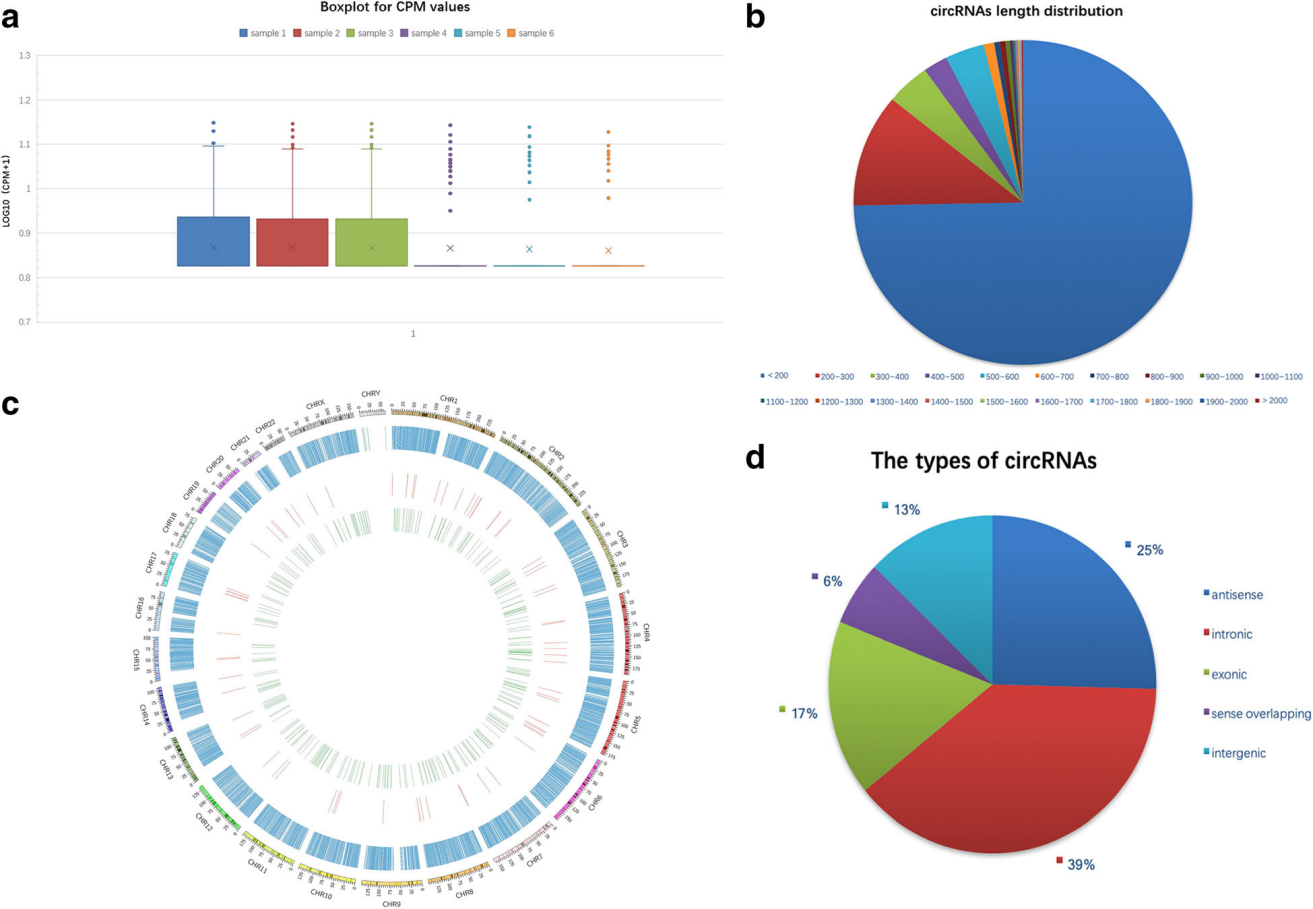
Overview of circRNAs in the PMOP and the control groups. (**a**) Expressions were normal in both the PMOP group (*samples 1-3*) and the control group (*samples 4-6*). (**b**) Length distribution of all circRNAs. (**c**) circRNAs were unevenly located in all chromosomes. (**d**) circRNAs were categorized into five types

In total, 8459 meaningful circRNAs were identified after mapping the sequencing reads to the human genome and abnormal expression was not observed in all six samples (Fig. 1a, Table 2). The sequence length, expression pattern, and distribution mapping of these circRNAs were assessed. The length of these circRNAs ranged from 106 to 94088 bp, with a frequent distribution length of < 200 bp (75%) and 200-300 bp (11%) (Fig. 1b). These circRNAs were unevenly located in all chromosomes, especially on chr1, chr2, chr3, and chr5 (Fig. 1c). They were categorized into five types based on their functions, including intronic, antisense, intergenic, exonic, and sense-overlapping circRNAs. Specifically, intronic cirRNAs constituted the largest portion (39%), followed by 25% of antisense, 17% of exonic, 13% of intergenic, and only 6% of sense overlapping (Fig. 1d).

**Table 2:** Overview of sequencing and quality control of samples

Sample	Raw reads	Clean reads	Mapped reads	Q30 (%)	OD260/280 ratio	Size (bp)	Total amount (ng)^b^
P1	71,752,862	70,830,830	50,966,866	90.56	1.84^a^	317	38.6
P2	81,810,500	80,354,146	59,939,550	90.48	1.92^a^	307	41.9
P3	81,942,214	80,005,894	55,444,504	90.66	1.82^a^	300	33.6
C1	80,311,782	79,697,224	61,590,054	91.77	1.85^a^	309	29.4
C2	90,455,988	89,212,696	68,539,836	91.95	1.88^a^	299	36.6
C3	81,985,938	81,104,462	62,490,876	91.86	1.81^a^	302	42.3

P1-3 represented samples of PMOF patients; C1-3 represented samples of healthy participates

^a^The O.D. A260/A280 ratio over 1.8 was acceptable for pure RNA

^b^The libraries were adjusted to 10 nM before cluster generation

**Fig. 2:**
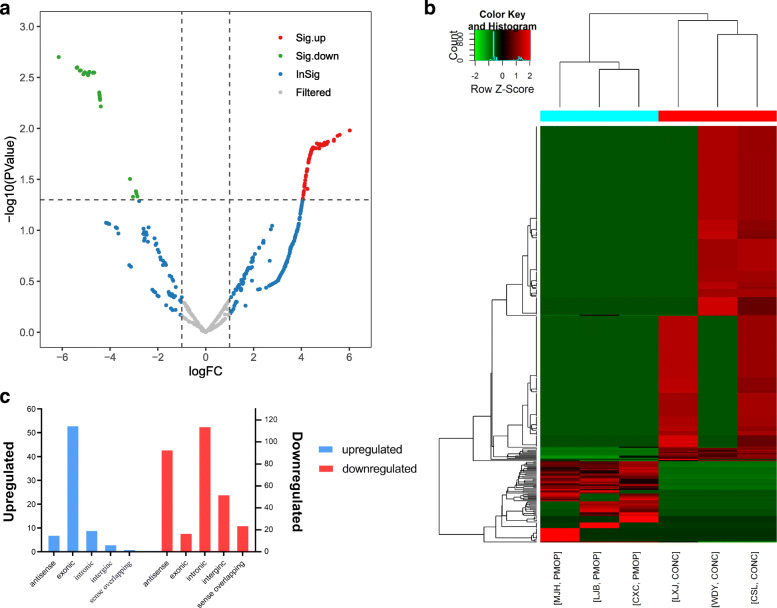
Comparison of differentially expressed circRNAs (DECs) between the PMOP and the control groups. (**a**) Distribution of DECs was exhibited via volcano plot map and compared between the PMOP group (*the former three columns*) and control group (*the latter three columns*). (**b**) Each row represented one circRNA and each column represented each sample. (**c**) DECs with different functions were exhibited

A hierarchical clustering approach was applied to determine the consistency of all samples. Volcano plot and heat-map analysis were used to identify DECs (Fig. 2a, b). Strikingly, 373 potential DECs comprising 123 intronic, 100 antisense, 70 exonic, 55 intergenic, and 25 sense-overlapping circRNAs were identified (Fig. 2c). Among these, 73 circRNAs were significantly upregulated and 300 were significantly downregulated in PMOP patients compared to the control individuals. The top 15 upregulated and downregulated circRNAs are listed in Table 3.

**Table 3:** TOP 15 upregulated and downregulated circRNAs in PMOP patients

Upregulated circRNAs			Downregulated circRNAs		
circRNA	logFC	*P* value	circRNA	LogFC	*P* value
circ_N4BP2L2	5.600022	0.011535	circ_XLOC_005754	−6.147327	0.001991
circ_PTPN12	5.512782	0.011801	circ_NAALADL2	−5.398425	0.002536
circ_MEMO1	5.359234	0.012858	circ_RP11-384J4.2	−5.369180	0.002507
circ_UIMC1	5.357413	0.013215	circ_CNTNAP5	−5.266750	0.002701
circ_ASAP1	5.105529	0.013469	circ_G012018	−5.262548	0.002704
circ_RAP1B	5.100567	0.013516	circ_GCLC	−5.242200	0.002700
circ_RASA1	5.069900	0.013857	circ_G013030	−5.225815	0.002694
circ_GNB1	5.057663	0.014177	circ_G075857	−5.175875	0.001938
circ_SIRT5	4.972721	0.014502	circ_ATP10A	−5.132593	0.002934
circ_PTK2	4.966612	0.013777	circ_CTD-2037K23.2	−5.115575	0.002338
circ_ACVR2A	4.896092	0.014309	circ_G059442	−5.105205	0.002914
circ_LRBA	4.891638	0.014695	circ_RP11-74E22.4	−5.101244	0.001915
circ_MBOAT2	4.807113	0.014602	circ_GPM6A	−5.095205	0.003121
circ_TTC27	4.771072	0.014249	circ_RP11-654C22.2	−5.085177	0.002879
circ_UIMC1	4.764548	0.014195	circ_G037579	−5.081723	0.008720

*logFC* log_2_Fold change

### qRT-PCR validation of DECs

To verify the reliability of sequencing data, four upregulated circRNAs (circ_0000471, circ_0008139, circ_0001824, circ_0008345) and four downregulated circRNAs (circ_0112054, circ_0000443, circ_0077548, circ_0001395) were randomly selected. The expression levels of these dysregulated circRNAs were following the sequencing data (Supplementary Figure 1).

### Functional annotation of DECs

**Fig. 3:**
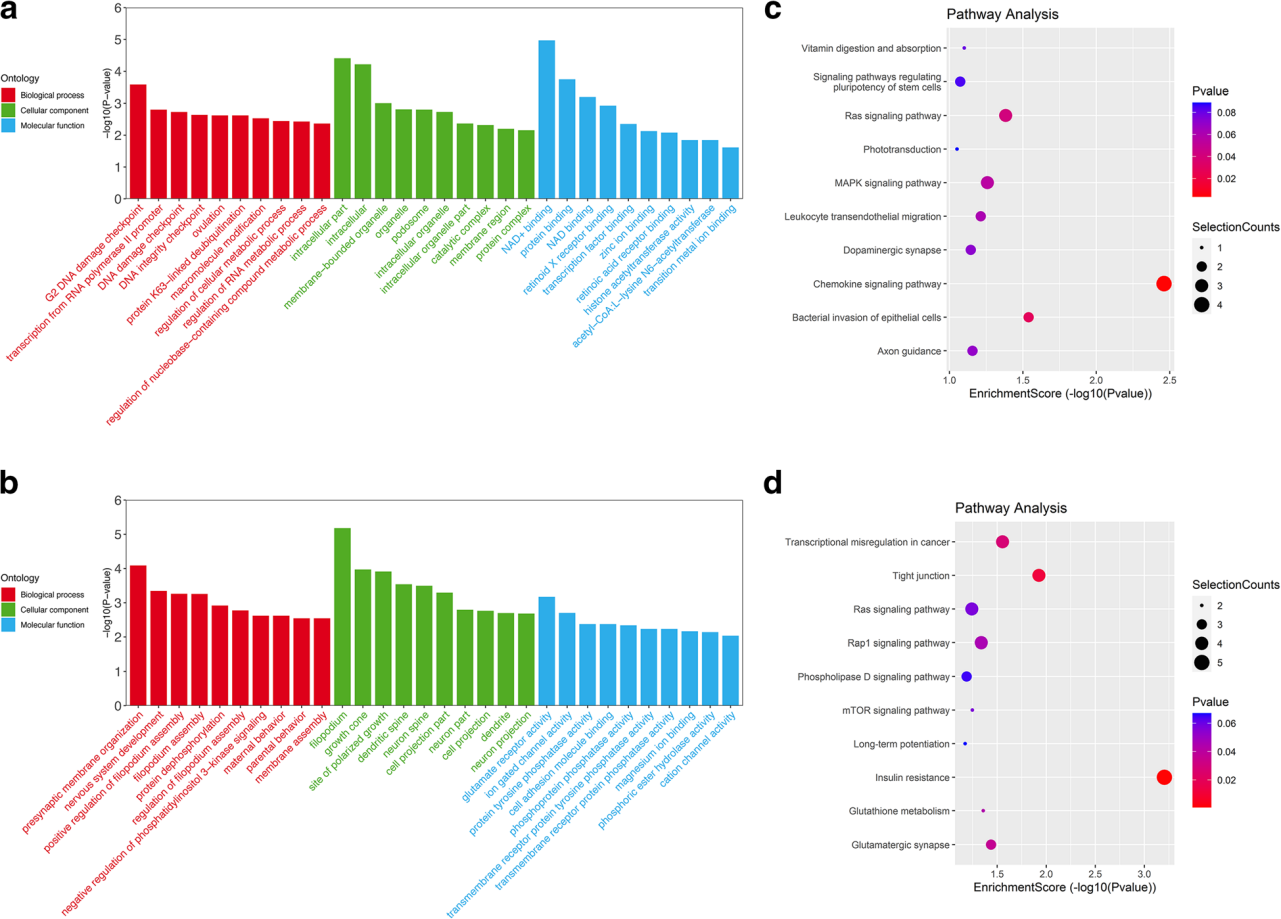
GO annotation and KEGG pathway analysis for DECs. GO analysis results of (**a**) upregulated and (**b**) downregulated circRNAs in terms of “biological process,” “cellular component,” and “molecular function.” TOP 10 pathway analysis results of (**c**) upregulated and (**d**) downregulated circRNAs based on the KEGG database

To explore the potential functions of these DECs, GO annotation and KEGG pathway analyses were separately performed with target coding genes of significantly upregulated and downregulated circRNAs. GO analysis comprised three elements, including the “cellular component,” “biological process,” and “molecular function.” The top 10 dysregulated GO processes of each subgroup were analyzed based on the dysregulated, enriched circRNAs derived from gene annotation. The upregulated circRNAs were found to be mostly enriched as follows: G2 DNA damage checkpoint, transcription from RNA polymerase II promoter, and DNA damage checkpoint in the “biological process” subgroup; intracellular part, intracellular and membrane-bounded organelle in the “cellular component” subgroup; and nicotinamide adenine dinucleotide (NAD+) binding, protein binding, and NAD binding in the “molecular function” subgroup (Fig. 3a, Supplementary Table 2). The significantly downregulated circRNAs were found to be mostly enriched as follows: presynaptic membrane organization, nervous system development, and positive regulation of filopodium assembly in the “biological process” subgroup; filopodium, growth cone, and site of polarized growth in the “cellular component” subgroup; and glutamate receptor activity, ion-gated channel activity, and protein tyrosine phosphatase activity in the “molecular function” subgroup (Fig. 3b, Supplementary Table 3). Furthermore, the top 20 KEGG pathways in the dysregulated circRNAs are shown in Fig. 3c and d (Supplementary Tables 4 and 5). The results revealed that significantly upregulated circRNAs were mainly associated with chemokine signaling pathway (hsa04062), bacterial invasion of epithelial cells (hsa05100), and RAS signaling pathway (hsa04014), while significantly downregulated circRNAs were mainly associated with insulin resistance (hsa04931), tight junction (hsa04530), and transcriptional misregulation (hsa05202).

### circRNA-miRNA co-expression analysis and miRNA-mRNA prediction

**Fig. 4:**
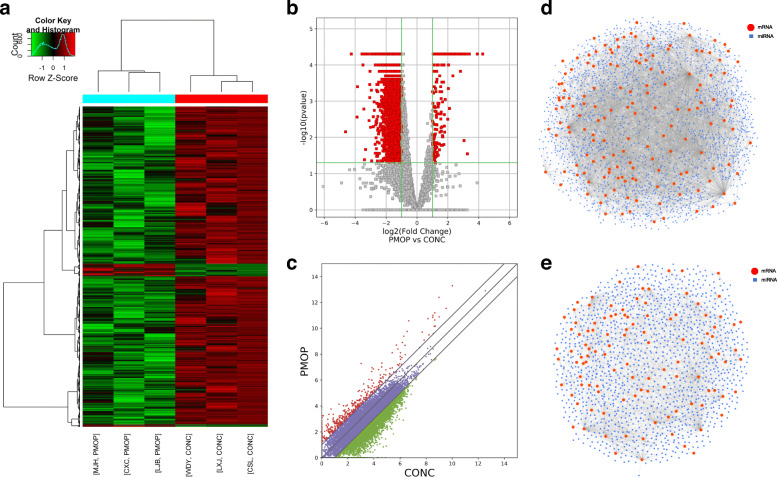
Differentially expressed mRNAs (DEMs) and prediction of miRNA-mRNA interactions. DEMs between the PMOP and the control groups were assessed and exhibited via (**a**) heat map, (**b**) volcano plot map, and (**c**) scatter plot analysis. Predicted interactions between (**d**) miRNAs (*blue square*) and upregulated mRNAs (*red circle*) and (**e**) downregulated mRNAs

A predicted circRNA-miRNA network comprising 171 nodes and 150 edges was constructed based on the top 15 DECs. It revealed that a single circRNA could target multiple miRNAs and vice versa (Supplementary Figure 2). Certain miRNAs might contribute to the abnormal expression of mRNAs in PMOP patients (Fig. 4a-c). Predicted miRNA-mRNA interactions were also visualized (Fig. 4d and e).

### Construction of competing endogenous RNA (ceRNA) network

**Fig. 5:**
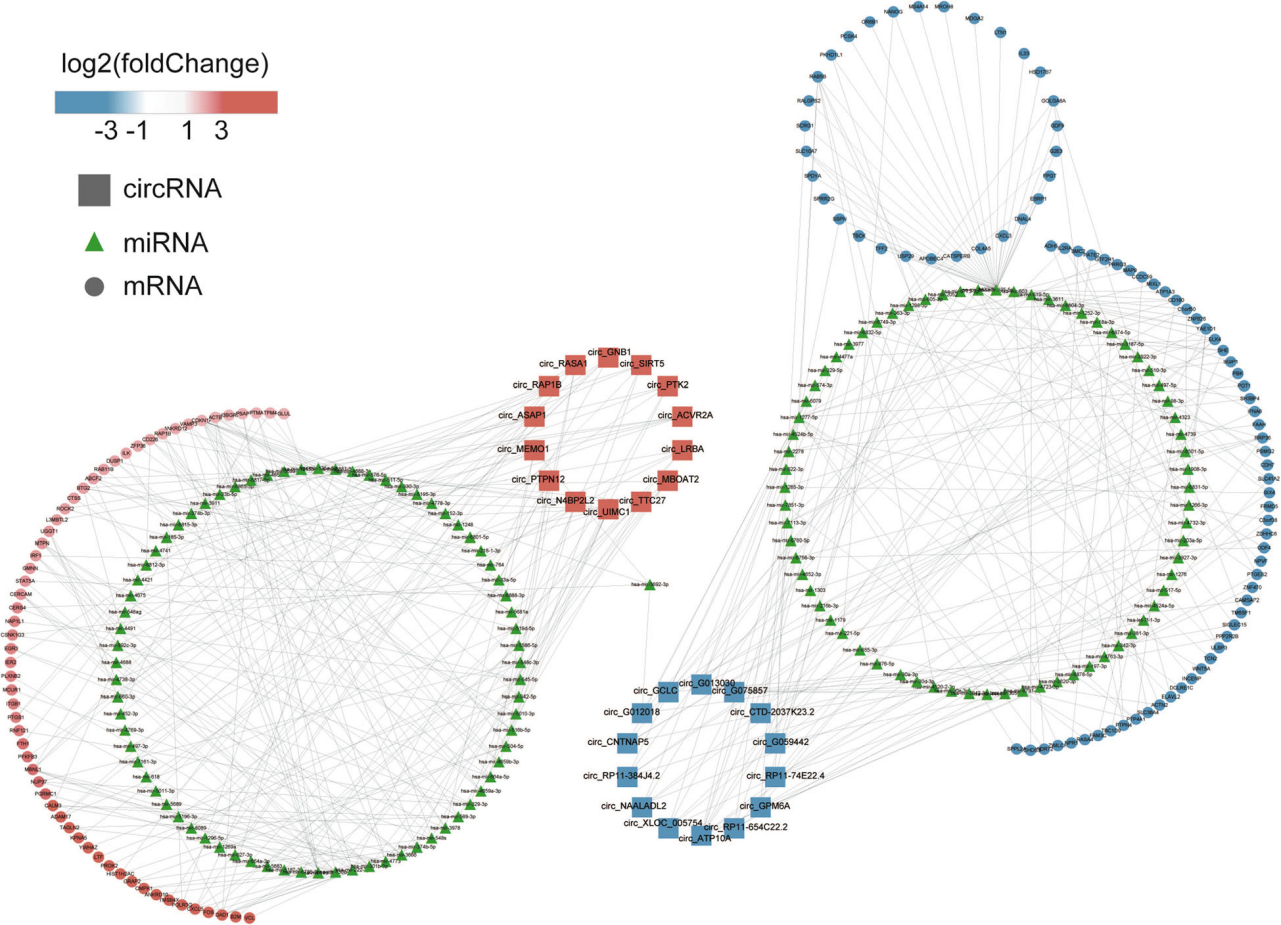
The ceRNA network was constructed based on circRNA-miRNA-mRNA interactions. Square, triangle, and circle nodes represent circRNA, miRNA, and mRNA, respectively. Red color indicates upregulation and blue color indicates downregulation

We integrated circRNA-miRNA and miRNA-mRNA interactions to construct a circRNA-miRNA-mRNA network, which provided preliminary insight into the association between the top 28 dysregulated circRNAs, 145 intermediate miRNAs, and 175 DEMs (Fig. 5).

## Discussion

Due to the absence of 5′ caps and 3′ polyadenylated tails, circRNAs have been ignored in polyadenylated transcriptome studies for quite a long time. With the advent of high-throughput sequencing, computational biology, and biochemical methods, accumulating evidence has demonstrated the critical roles of circRNAs during the pathological process in various diseases [8, 11, 12]. Benefiting from covalently closed-loop structures, circRNAs could serve as promising diagnostic and prognostic BTMs ascribed to their relative tolerance to exonucleases [6, 11]. However, the role of circRNAs in PMOP and the mechanism by which circRNA regulates the balance between osteogenesis and osteoclastogenesis have not been well elucidated. Herein, we explored the DECs in PBMCs between PMOP patients and control individuals using whole transcriptome sequencing and further confirmed the expression level of these DECs by combining them with the public dataset. These findings are foundational for subsequent studies on circRNAs in PMOP and may offer insight into prevention and new treatment targets for PMOP.

A consensus has been reached that ideal biomarkers for the diagnosis of PMOP and therapeutic drug monitoring should have the characteristics of abundance, easy-access, and less-invasiveness. Firstly, in the clinical setting, a blood sample is typically the starting point for biomarker search and discovery. The extraction of blood has minimal risks when performed by a trained technician. PBMCs are a great source of DNA for genetic analysis, in vitro culturing, and functional assays or for subsequent isolation of lymphocyte or monocyte sub-types, provided appropriate cryopreservation [16]. Secondly, there is growing evidence that morphological and functional changes in PBMCs, in most lymphocytes and monocytes, may reflect the severity of osteoporosis in postmenopausal women [17, 18]. PBMCs are becoming a fairly common subject of research in the field of osteoporosis, and the ease of obtaining the material vs bone biopsy is an indisputable advantage [19, 20].

In the present study, 373 DECs, including 73 upregulated and 300 downregulated circRNAs, were identified between PMOP patients and paired healthy individuals. Strikingly, 8 circRNAs (hsa_circ_0000471, hsa_circ_0008139, hsa_circ_0007385, hsa_circ_0008631, hsa_circ_0001824, hsa_circ_0027464, hsa_circ_0007507, hsa_circ_0000008) were significantly upregulated (|FC|>5) and 5 circRNAs (all novel) were significantly downregulated (|FC|>9), which implied that these DECs might exert crucial functions in regulating the pathogenesis and progression of PMOP. These DECs were mainly intronic and antisense circRNAs distributed among all chromosomes, with lengths mainly less than 300 bp. Then, GO annotation and KEGG pathway analyses were performed to elucidate the functions of DECs between PMOP and healthy individuals. The most significant GO items were G2 DNA damage checkpoint, transcription from RNA polymerase II promoter, NAD+ or protein binding, presynaptic membrane organization, and glutamate receptor activity, indicating that the coding genes contributed to the development of PMOP. In KEGG pathway analysis, several important pathways, including hsa04931 (insulin resistance), hsa04062 (chemokine signaling pathway), and has04530 (tight junction), were identified to take pivotal parts in the pathogenesis of PMOP. For instance, insulin resistance might affect osteoclast differentiation, activation, and survival via the tumor necrosis factor-related cytokine receptor activator of nuclear factor kappa B ligand (RANKL)-induced pathway, which aggravated the osteoporosis process in postmenopausal women [21]. Moreover, activation of C-C chemokine receptor-2 (CCR2) is reported to be crucially involved in the signaling of nuclear factor-kappa B (NF-κB) and extracellular signal-related kinase 1 and 2 (ERK1/2), which contribute to the susceptibility to RANKL-induced osteoclastogenesis [22].

As highly conserved endogenous non-coding RNAs, numerous circRNAs harbor numerous miRNA binding sites, suggesting that they could sponge certain miRNAs and function as ceRNAs to regulate gene expression in several diseases [23–26]. circNT5E, a circRNA formed from the NT5E genome and regulated by the RNA-editing enzyme, was reported to exert vital regulatory function by sponging glioblastoma suppressor miR-422a [27]. circNHSL1 was also reported to intervene in gastric cancer progression by sponging miR-1306-3p and could serve as a novel biomarker for early diagnosis of gastric cancer [28]. Similarly, highly expressed circ-SFMBT2 was observed in gastric cancer tissues and proved to participate in the pathogenesis of gastric cancer via miR-182-5p sponging [29]. To date, only a few pathogenic circRNAs have been reported in osteoporosis and RNA sequencing tools and bioinformatics analysis have not been employed in these studies [30–32]. Herein, we constructed ceRNA networks with DECs and DEMs from whole transcriptome sequencing data, as well as intermediate miRNAs predicted through bioinformatics analysis. These data verified that circRNAs might exert regulatory functions in PMOP through miRNA response elements (MREs), which facilitated our understanding of the pathogenesis of PMOP.

An unavoidable limitation of this study was the relatively small sample size, which was ascribed to the strict inclusion and exclusion criteria. However, the demographic data of all samples were well balanced, which was beneficial to reduce system error from high-throughput sequencing and lower false-positive results. Nevertheless, a prospective study with a larger sample size and more validation techniques is ongoing.

## Conclusions

The present study investigated potential circRNA-mediated ceRNA interplays using sample-matched whole transcriptome profiles between PMOP patients and healthy individuals. This PMOP-specific dysregulated ceRNA network might provide a comprehensive understanding of ceRNA-mediated gene regulation in the pathogenesis of PMOP and lay a firm foundation for exploring promising diagnostic biomarkers and novel treatment targets for PMOP.

## Supplementary Information

**Additional file 1: Supplementary Excel 1**. ceRNA network interactions.

**Additional file 2: Supplementary Figure 1**. qRT-PCR verification of the expression levels of the four upregulated circRNAs (a) and four downregulated circRNAs (b).

**Additional file 3: Supplementary Figure 2**. Predicted circRNA-miRNA network comprising 171 nodes and 150 edges.

**Additional file 4: Supplementary Table 1**. Primers for qRT-PCR validation of DECs.

**Additional file 5: Supplementary Table 2**. Significantly enriched GO terms based on upregulated DECs.

**Additional file 6: Supplementary Table 3**. Significantly enriched GO terms based on downregulated DECs.

**Additional file 7: Supplementary Table 4**. Top 10 KEGG pathways based on upregulated DECs.

**Additional file 8: Supplementary Table 5**. Top 10 KEGG pathways based on downregulated DECs.

## Data Availability

The data analyzed during the study are available from the corresponding author on reasonable request.

## Abbreviations

PMOP: Postmenopausal osteoporosis
circRNAs: Circular RNAs
DECs: Differentially expressed circRNAs
PBMCs: Peripheral blood mononuclear cells
GO: Gene Ontology
KEGG: Kyoto Encyclopedia of Genes and Genomes
BTMs: Bone turnover markers
ncRNAs: Non-coding RNAs
miRNAs: microRNAs
mRNA: messenger RNA
PKP: Percutaneous kyphoplasty
BMD: Bone mineral density
FPKM: Fragments per kilobase of transcript per million fragments mapped
FC: Fold-change
DAVID: Database for Annotation, Visualization and Integrated Discovery
RANKL: Receptor activator of nuclear factor kappa B ligand
CCR2: C-C chemokine receptor-2
NF-κB: Nuclear factor-kappa B
ERK1/2: Extracellular signal-related kinase 1 and 2
MREs: miRNA react elements
