# Supplementary figures and images for: Identification and comparison of novel circular RNAs with associated co-expression and competing endogenous RNA networks in postmenopausal osteoporosis
Source: J Orthop Surg Res. 2021 Jul 16;16:459. doi: 10.1186/s13018-021-02604-1 (PMC8285836; doi:10.1186/s13018-021-02604-1)

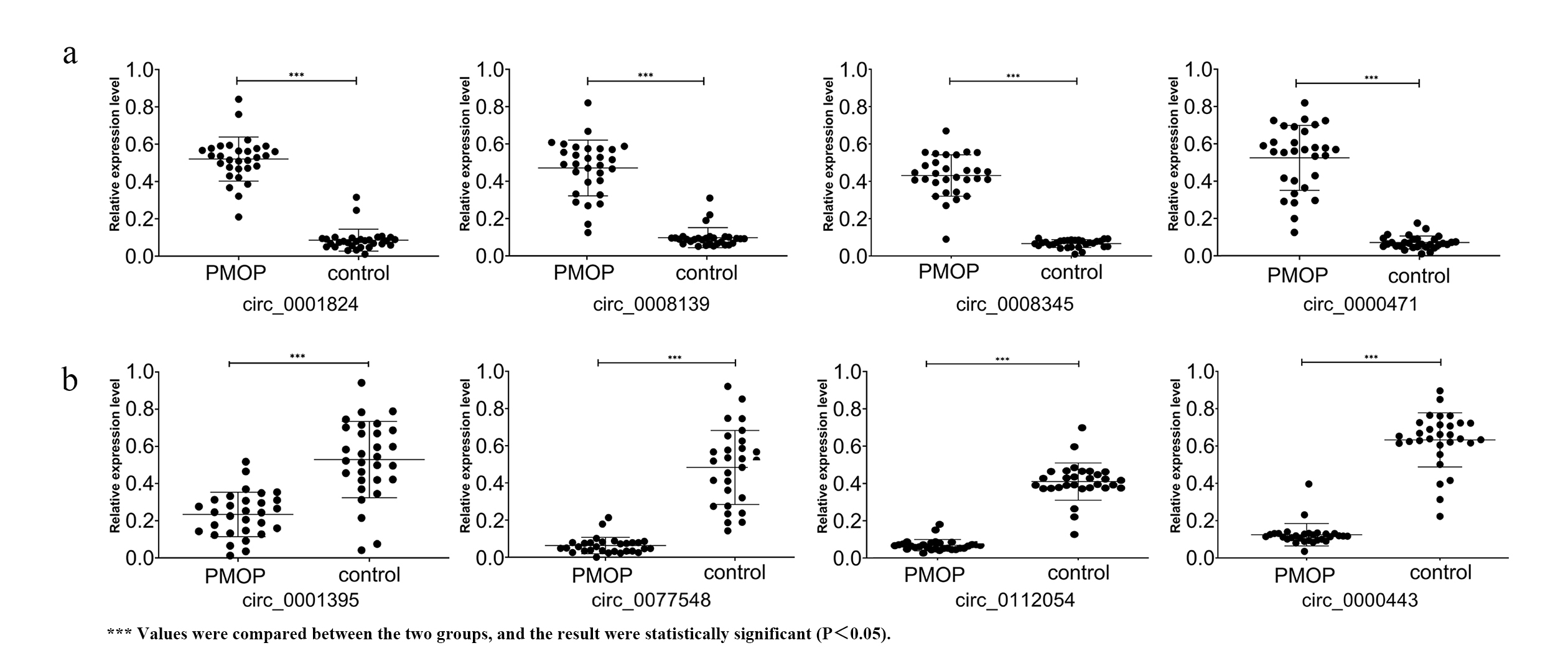

Supplement: Supplementary file 2 — Additional file 2: Supplementary Figure 1. qRT-PCR verification of the expression levels of the four upregulated circRNAs (a) and four downregulated circRNAs (b). [file 13018_2021_2604_MOESM2_ESM.jpg]

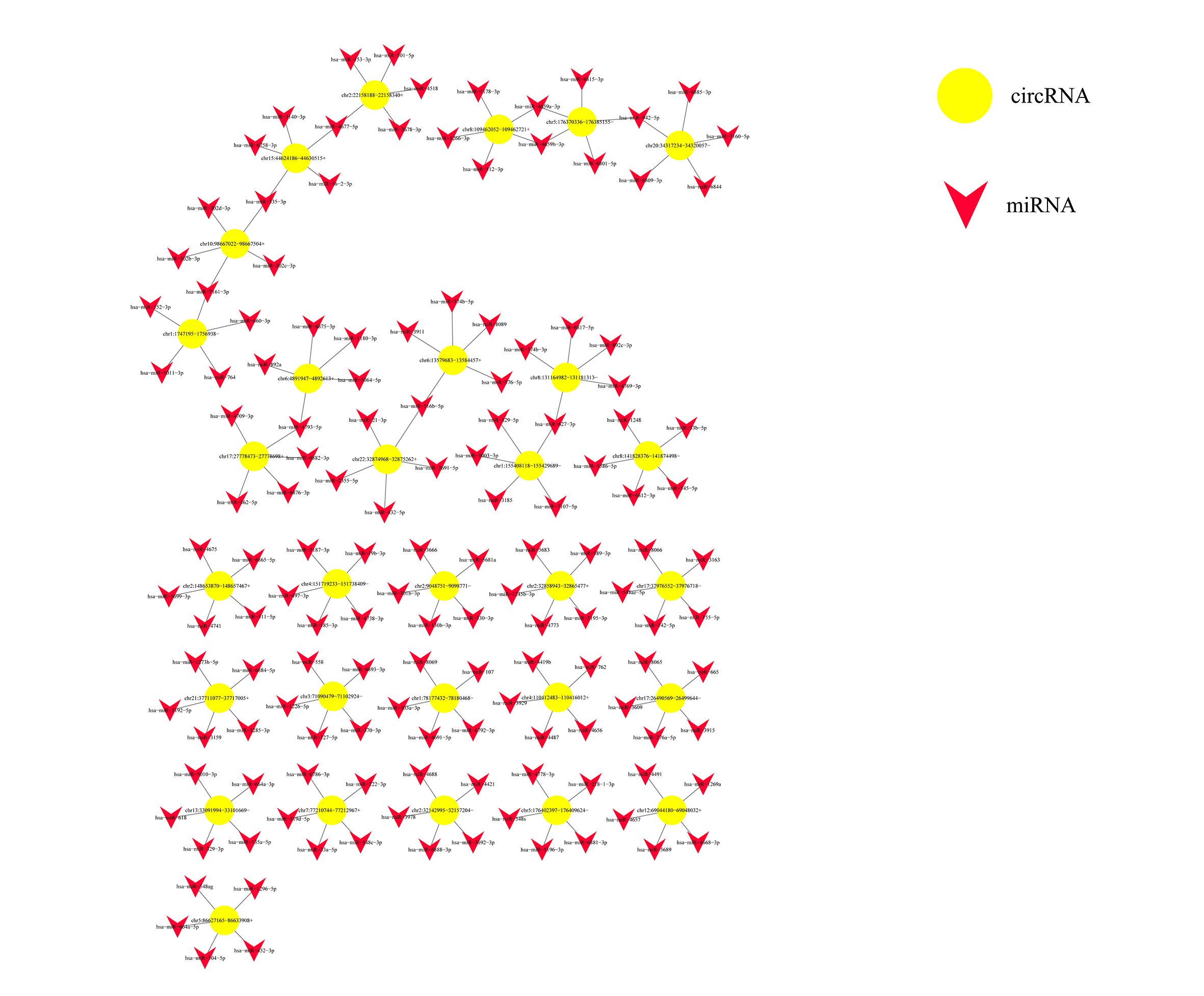

Supplement: Supplementary file 3 — Additional file 3: Supplementary Figure 2. Predicted circRNA-miRNA network comprising 171 nodes and 150 edges. [file 13018_2021_2604_MOESM3_ESM.jpg]
