# Supplementary material for: Identification and comparison of novel circular RNAs with associated co-expression and competing endogenous RNA networks in postmenopausal osteoporosis
Source: J Orthop Surg Res. 2021 Jul 16;16:459. doi: 10.1186/s13018-021-02604-1 (PMC8285836; doi:10.1186/s13018-021-02604-1)
Supplement: Supplementary file 4 — Additional file 4: Supplementary Table 1. Primers for qRT-PCR validation of DECs. [file 13018_2021_2604_MOESM4_ESM.doc]

Supplementary Table 1. Primers for qRT-PCR validation of DECs.

| CircRNA | | Sequence (5’ to 3’) |
| --- | --- | --- |
| circ_0000471 | forward | CAAAGACCTCCTCCTCCACA |
|  | reverse | TGCCATCACGATTCTGACCA |
| circ_0008139 | forward | GCAACTCAAGGACCTTTAGCA |
|  | reverse | TGTCAATTTAACTCGGCTGTGA |
| circ_0001824 | forward | CCGGAAAGTATGGCAGAGGA |
|  | reverse | TCAGCTGTTTTCAAGCCATCT |
| circ_0008345 | forward | TGAGCTTGTGAGTGAGTGGT |
|  | reverse | GCAAGGAGAATGGCGAGATG |
| circ_0112054 | forward | GCTGGATGAGCAAGATGGTG |
|  | reverse | TTGCAGCCATTCCAGAGAGA |
| circ_0077548 | forward | TGATGAAGCAGAGTGGCAAG |
|  | reverse | TCTTCTTCAGAATCACTGCCCT |
| circ_0001395 | forward | AGAGGCTGTCAGAAAGCTGG |
|  | reverse | CTGCCCGTCTAACTTCTGGA |
| circ_0001147 | forward | TCGTGGGATCTTTGAGCCTT |
|  | reverse | CCCTTGGTCGAATTCTTGCC |
| β－actin | forward | CAGGGCGTGATGGTGGGCA |
|  | reverse | CAAACATCATCTGGTCATCTTC |
