# Supplementary material for: Identification and comparison of novel circular RNAs with associated co-expression and competing endogenous RNA networks in postmenopausal osteoporosis
Source: J Orthop Surg Res. 2021 Jul 16;16:459. doi: 10.1186/s13018-021-02604-1 (PMC8285836; doi:10.1186/s13018-021-02604-1)
Supplement: Supplementary file 5 — Additional file 5: Supplementary Table 2. Significantly enriched GO terms based on upregulated DECs. [file 13018_2021_2604_MOESM5_ESM.docx]

Supplementary Table 2. Significantly enriched GO terms based on upregulated DECs.

| **GO ID** | **Term** | **Ontology** | **Fold Enrichment** | | | **P value** | **Enrichment Score** |
| --- | --- | --- | --- | --- | --- | --- | --- |
| GO:0031572 | G2 DNA damage checkpoint | Biological process | | 23.98660714 | 0.000261446 | | 3.582617432 |
| GO:0006366 | transcription from RNA polymerase II promoter | Biological process | | 2.377433628 | 0.00158364 | | 2.800343518 |
| GO:0000077 | DNA damage checkpoint | Biological process | | 7.624881741 | 0.001862282 | | 2.729954477 |
| GO:0031570 | DNA integrity checkpoint | Biological process | | 7.195982143 | 0.002299301 | | 2.638404241 |
| GO:0030728 | ovulation | Biological process | | 27.41326531 | 0.002386205 | | 2.622292194 |
| GO:0070536 | protein K63-linked deubiquitination | Biological process | | 27.41326531 | 0.002386205 | | 2.622292194 |
| GO:0043412 | macromolecule modification | Biological process | | 1.848085302 | 0.00295259 | | 2.52979688 |
| GO:0031323 | regulation of cellular metabolic process | Biological process | | 1.544659379 | 0.003596259 | | 2.444148994 |
| GO:0051252 | regulation of RNA metabolic process | Biological process | | 1.772097625 | 0.003739959 | | 2.427133109 |
| GO:0019219 | regulation of nucleobase-containing compound metabolic process | Biological process | | 1.652665755 | 0.004318748 | | 2.364642165 |
| GO:0044424 | intracellular part | Cellular component | | 1.287699702 | 3.91329E-05 | | 4.407458037 |
| GO:0005622 | intracellular | Cellular component | | 1.275559164 | 6.06313E-05 | | 4.217303101 |
| GO:0043227 | membrane-bounded organelle | Cellular component | | 1.299016153 | 0.000987758 | | 3.005349526 |
| GO:0043226 | organelle | Cellular component | | 1.251167539 | 0.001548079 | | 2.810206935 |
| GO:0002102 | podosome | Cellular component | | 33.68421053 | 0.001581083 | | 2.801045229 |
| GO:0043229 | intracellular organelle | Cellular component | | 1.288284001 | 0.001862403 | | 2.729926296 |
| GO:0044446 | intracellular organelle part | Cellular component | | 1.472533794 | 0.004293539 | | 2.367184585 |
| GO:1902494 | catalytic complex | Cellular component | | 2.9978531 | 0.004822137 | | 2.316760446 |
| GO:0098589 | membrane region | Cellular component | | 2.335249889 | 0.006311466 | | 2.199869772 |
| GO:0043234 | protein complex | Cellular component | | 1.686441269 | 0.006998391 | | 2.15500178 |
| GO:0070403 | NAD+ binding | Molecular function | | 67.14102564 | 1.07134E-05 | | 4.970072445 |
| GO:0005515 | protein binding | Molecular function | | 1.45749841 | 0.000179137 | | 3.746815085 |
| GO:0051287 | NAD binding | Molecular function | | 17.81292517 | 0.000632335 | | 3.199052879 |
| GO:0046965 | retinoid X receptor binding | Molecular function | | 38.79259259 | 0.001183104 | | 2.926976894 |
| GO:0008134 | transcription factor binding | Molecular function | | 3.853568801 | 0.004454003 | | 2.351249508 |
| GO:0008270 | zinc ion binding | Molecular function | | 2.406488374 | 0.007443959 | | 2.128196015 |
| GO:0042974 | retinoic acid receptor binding | Molecular function | | 14.54722222 | 0.008319182 | | 2.079919382 |
| GO:0004402 | histone acetyltransferase activity | Molecular function | | 10.97903564 | 0.014285323 | | 1.845109939 |
| GO:0090595 | acetyl-CoA:L-lysine N6-acetyltransferase | Molecular function | | 10.97903564 | 0.014285323 | | 1.845109939 |
| GO:0046914 | transition metal ion binding | Molecular function | | 2.007898167 | 0.024263249 | | 1.61505105 |
