# Supplementary material for: Identification and comparison of novel circular RNAs with associated co-expression and competing endogenous RNA networks in postmenopausal osteoporosis
Source: J Orthop Surg Res. 2021 Jul 16;16:459. doi: 10.1186/s13018-021-02604-1 (PMC8285836; doi:10.1186/s13018-021-02604-1)
Supplement: Supplementary file 6 — Additional file 6: Supplementary Table 3. Significantly enriched GO terms based on downregulated DECs. [file 13018_2021_2604_MOESM6_ESM.docx]

Supplementary Table 3. Significantly enriched GO terms based on downregulated DECs.

| **GO ID** | **Term** | **Ontology** | **Fold Enrichment** | **P value** | **Enrichment**  **Score** |
| --- | --- | --- | --- | --- | --- |
| GO:0097090 | presynaptic membrane organization | Biological process | 34.12632322 | 8.18874E-05 | 4.086782758 |
| GO:0007399 | nervous system development | Biological process | 1.975796034 | 0.000451869 | 3.344987409 |
| GO:0051491 | positive regulation of filopodium assembly | Biological process | 18.48509174 | 0.000548957 | 3.260461706 |
| GO:0046847 | filopodium assembly | Biological process | 10.56290957 | 0.000554437 | 3.256147806 |
| GO:0006470 | protein dephosphorylation | Biological process | 5.070196592 | 0.001209214 | 2.917496996 |
| GO:0051489 | regulation of filopodium assembly | Biological process | 12.67549148 | 0.001681859 | 2.774210498 |
| GO:0014067 | negative regulation of phosphatidylinositol 3-kinase signaling | Biological process | 26.88740617 | 0.002394756 | 2.620738768 |
| GO:0042711 | maternal behavior | Biological process | 26.88740617 | 0.002394756 | 2.620738768 |
| GO:0060746 | parental behavior | Biological process | 24.64678899 | 0.002861047 | 2.54347507 |
| GO:0071709 | membrane assembly | Biological process | 24.64678899 | 0.002861047 | 2.54347507 |
| GO:0030175 | filopodium | Cellular component | 13.03003645 | 6.62383E-06 | 5.178890649 |
| GO:0030426 | growth cone | Cellular component | 7.993215635 | 0.000106813 | 3.971377762 |
| GO:0030427 | site of polarized growth | Cellular component | 7.796661152 | 0.000122535 | 3.911739282 |
| GO:0043197 | dendritic spine | Cellular component | 8.615875548 | 0.000289606 | 3.538191817 |
| GO:0044309 | neuron spine | Cellular component | 8.432559047 | 0.000320049 | 3.494783022 |
| GO:0044463 | cell projection part | Cellular component | 2.915017583 | 0.000506951 | 3.295034149 |
| GO:0097458 | neuron part | Cellular component | 2.457862172 | 0.001610281 | 2.793098286 |
| GO:0042995 | cell projection | Cellular component | 2.075885659 | 0.001729103 | 2.762179078 |
| GO:0030425 | dendrite | Cellular component | 3.52293578 | 0.002008364 | 2.697157673 |
| GO:0043005 | neuron projection | Cellular component | 2.620365456 | 0.002077782 | 2.682400055 |
| GO:0008066 | glutamate receptor activity | Molecular function | 17.28382838 | 0.000674245 | 3.171182002 |
| GO:0022839 | ion gated channel activity | Molecular function | 11.96572734 | 0.001992242 | 2.700657977 |
| GO:0004725 | protein tyrosine phosphatase activity | Molecular function | 6.100174723 | 0.004224298 | 2.374245431 |
| GO:0050839 | cell adhesion molecule binding | Molecular function | 4.713771377 | 0.004235717 | 2.373073039 |
| GO:0004721 | phosphoprotein phosphatase activity | Molecular function | 4.629596888 | 0.004570823 | 2.340005556 |
| GO:0005001 | transmembrane receptor protein tyrosine phosphatase activity | Molecular function | 17.28382838 | 0.005854407 | 2.232517061 |
| GO:0019198 | transmembrane receptor protein phosphatase activity | Molecular function | 17.28382838 | 0.005854407 | 2.232517061 |
| GO:0000287 | magnesium ion binding | Molecular function | 4.204174472 | 0.006834756 | 2.165276981 |
| GO:0042578 | phosphoric ester hydrolase activity | Molecular function | 3.120003404 | 0.007267444 | 2.138618319 |
| GO:0005261 | cation channel activity | Molecular function | 3.345257106 | 0.009234217 | 2.034599935 |
