# Supplementary material for: Identification and comparison of novel circular RNAs with associated co-expression and competing endogenous RNA networks in postmenopausal osteoporosis
Source: J Orthop Surg Res. 2021 Jul 16;16:459. doi: 10.1186/s13018-021-02604-1 (PMC8285836; doi:10.1186/s13018-021-02604-1)
Supplement: Supplementary file 7 — Additional file 7: Supplementary Table 4. Top 10 KEGG pathways based on upregulated DECs. [file 13018_2021_2604_MOESM7_ESM.docx]

Supplementary Table 4. Top 10 KEGG pathways based on upregulated DECs.

| **Pathway ID** | **Definition** | **Count** | **Enrichment_Score** |
| --- | --- | --- | --- |
| hsa04062 | Chemokine signaling pathway | 187 | 2.460365 |
| hsa05100 | Bacterial invasion of epithelial cells | 78 | 1.538326 |
| hsa04014 | Ras signaling pathway | 227 | 1.383115 |
| hsa04010 | MAPK signaling pathway | 255 | 1.258033 |
| hsa04670 | Leukocyte transendothelial migration | 118 | 1.212762 |
| hsa04360 | Axon guidance | 127 | 1.156701 |
| hsa04728 | Dopaminergic synapse | 129 | 1.144862 |
| hsa04977 | Vitamin digestion and absorption | 24 | 1.100422 |
| hsa04550 | Signaling pathways regulating pluripotency of stem cells | 142 | 1.072747 |
| hsa04744 | Phototransduction | 27 | 1.051391 |
