# Supplementary material for: Identification and comparison of novel circular RNAs with associated co-expression and competing endogenous RNA networks in postmenopausal osteoporosis
Source: J Orthop Surg Res. 2021 Jul 16;16:459. doi: 10.1186/s13018-021-02604-1 (PMC8285836; doi:10.1186/s13018-021-02604-1)
Supplement: Supplementary file 8 — Additional file 8: Supplementary Table 5. Top 10 KEGG pathways based on downregulated DECs. [file 13018_2021_2604_MOESM8_ESM.docx]

Supplementary Table 5. Top 10 KEGG pathways based on downregulated DECs.

| **Pathway ID** | **Definition** | **Count** | **Enrichment_Score** |
| --- | --- | --- | --- |
| hsa04931 | Insulin resistance | 109 | 3.203872 |
| hsa04530 | Tight junction | 139 | 1.925449 |
| hsa05202 | Transcriptional misregulation in cancer | 180 | 1.554561 |
| hsa04724 | Glutamatergic synapse | 114 | 1.437434 |
| hsa00480 | Glutathione metabolism | 52 | 1.35803 |
| hsa04015 | Rap1 signaling pathway | 211 | 1.338427 |
| hsa04150 | mTOR signaling pathway | 60 | 1.246557 |
| hsa04014 | Ras signaling pathway | 227 | 1.242492 |
| hsa04072 | Phospholipase D signaling pathway | 144 | 1.187916 |
| hsa04720 | Long-term potentiation | 66 | 1.173537 |
